# Propagation of Sounds through Small Panels Made of Polymer Materials by 3D Printing

**DOI:** 10.3390/polym16010005

**Published:** 2023-12-19

**Authors:** Adelina Hrițuc, Andrei Marius Mihalache, Oana Dodun, Gheorghe Nagîț, Irina Beșliu-Băncescu, Bruno Rădulescu, Laurențiu Slătineanu

**Affiliations:** 1 Department of Machine Manufacturing Technology, “Gheorghe Asachi” Technical University of Iași, 700050 Iasi, Romania; andrei.mihalache@tuiasi.ro (A.M.M.); oanad@tcm.tuiasi.ro (O.D.); nagit@tcm.tuiasi.ro (G.N.); slati@tcm.tuiasi.ro (L.S.); 2 Faculty of Mechanical Engineering, Automotive, and Robotics, “Stefan cel Mare” University, 720229 Suceava, Romania; irina.besliu@usm.ro; 3 Department of Machine Tools, “Gheorghe Asachi” Technical University of Iași, 700050 Iasi, Romania; bruno.radulescu@academic.tuiasi.ro

**Keywords:** sound propagation, polymeric material, small panel, factorial experiment, influencing factors, empirical mathematical model

## Abstract

To evaluate the sound insulation capacity of small panels made of polymeric materials by 3D printing, a Taguchi L18-type factorial experiment with eight independent variables was designed and materialized. The independent variables were the panel thickness, polymer material type, 3D printing speed, infill percent, infill pattern, layer thickness, frequency, and sound volume. Empirical mathematical models were determined through the mathematical processing of the experimental results using specialized software. These empirical mathematical models highlight the meaning and intensity of the influence exerted by the input factors in the process on the acoustic pressure level of the energy absorbed after the passage of sounds through the small panels manufactured by 3D printing from polylactic acid and polyethylene terephthalate glycol. The factor with the strongest influence was the frequency of the sounds, with a maximum of the sound pressure level for a frequency of 13,000 Hz. A polylactic acid panel between the sound source and the sound-receiving sensor reduces the sound pressure level by about 45% from 95.8 to 65.8 dB. The power function type mathematical model in the case of the energy absorbed by the panel highlights the fact that the highest values of the exponents are those attached to the sound frequency (exponent equal to 1.616) and, respectively, to the thickness of the panel (exponent equal to −0.121).

## 1. Introduction

Sounds are vibrations that travel through an elastic medium and can be sensed by the human ear. This means that the vibrations corresponding to the sounds must have a certain intensity and, respectively, a certain frequency that makes it possible to sense them via a membrane in the human ear. Intensity and frequency are two essential characteristics of sounds, to which, from a musical point of view, duration and timbre are added. Unlike vibrations co-responsive to sounds, noises are vibrations less desired or agreed upon by human beings.

In general, sounds have a frequency between 16 Hz and 20 kHz, but it is also possible for some people to perceive frequencies slightly lower than 16 Hz, just as other people can perceive sounds with frequencies slightly higher than 20 kHz. With age, the ability of the human ear to detect sounds characterized by high frequencies decreases.

Using sounds, people can communicate. They can detect the proximity of vehicles, the presence of other beings, or natural or artificial systems capable of generating or reflecting sounds.

It is normal for sounds to be absorbed by different media, as there may be situations where sounds are amplified.

**Figure 1:**
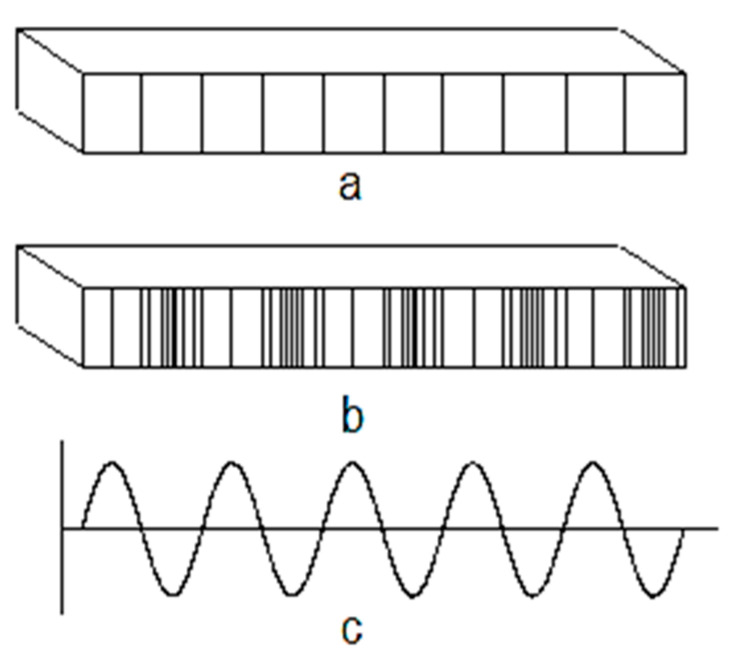
Propagation of sounds through a bar with a square section of solid material, highlighted by the time displacement of some elongations and contractions: (**a**) bar unaffected by sound propagation; (**b**) bar through which elongations and contractions corresponding to the sound are propagated; (**c**) time variation of elongations and contractions.

It is known that sounds propagate at speeds that differ from the constitution of the environment they travel through. Thus, the sound propagation speed can have distinct values in different environments. If sounds propagate in the air with speeds of 330–350 m/s, in water, the sound propagation speed can be 1–4 m/s, while in a steel piece, the sound speed can have values of 5100 to 5900 m/s. In parts made of solid materials, sounds propagate through successive elongations and contractions of some areas of the part (Figure 1).

The situation in which the absorption of sound energy by the environment is necessary occurs mainly when the problem of so-called sound insulation is raised, when technical measures are taken that are mainly aimed at reducing the intensity of sounds. In such a case, the problem of identifying and determining the characteristics of materials with sound insulation properties arises.

The emergence of 3D printing processes has led, among other things, to the need to evaluate the ability of materials from 3D printed parts to be used as insulating materials from a sound point of view, and such a problem has been addressed by researchers in the field of manufacturing parts from polymeric materials by 3D printing.

Thus, Alfarisi et al. (2021) investigated the problem of improving the quality of sounds in the case of a music box for which some components were made by 3D printing [1]. They used the finite element method to identify possibilities for improving the materials’ ability to transmit sounds.

King et al. have addressed the problem of studying the ability of some panels made by 3D printing to absorb noise using a sound impedance tube [2]. They found that a model characterized by a micro-perforated design can absorb noises characterized by a frequency between 100 and 6400 Hz.

Vasina et al. studied the sound absorption properties of open-porous acrylonitrile butadiene styrene structures manufactured by 3D printing technologies [3]. One of their conclusions was that the sound absorption properties of the samples made from the investigated material could be significantly influenced by the type of 3D printing process, the structure of the material, the frequency of excitation, the thickness of the sample, and the size of the interstice in the back of the absorbent material panel.

Hrițuc et al. proposed using equipment to study sound propagation through small panels manufactured by 3D printing [4,5]. They researched the influence of different factors on the sound pressure level after passing sounds through such panels.

The experimental research carried out has shown that the main factors or groups of factors able to influence the sound propagation capacity of some materials incorporated in parts manufactured by 3D printing are the following:

- The nature of the sample material [3,6,7,8];
- The proportion of different materials in the sample [6];
- The structure corresponding to the arrangement of the material in parts [2,3,9,10,11,12,13,14,15,16,17,18,19,20];
- Imperfections in the sample material [14,17];
- The size of the so-called “throat effect” (the throat is the smallest aperture in a series of interconnected pores) [9,10,21];
- Test sample manufacturing technology [7,14,17];
- The shape of the cavity in which the testing is performed [22];
- The frequency of sounds that pass through parts manufactured by 3D printing [3,5,6,8,9,12,14,15,20,22];
- The acoustic pressure level [5];
- The distance from the test sample to the sound receiver [3,12,15];
- Sample thickness [3,5,13,15,23];
- The parameters corresponding to the 3D printing process [5,7], etc.

Sounds are generated by operating various categories of equipment, by a loudspeaker or by the voice boxes of humans or other animals, by the rubbing of wings by some insects, etc. For example, the operation of a crank-rod mechanism, an internal combustion engine, an electric motor, a percussive hammer, etc., is accompanied by the generation of vibrations that, if they have a certain intensity and a frequency within certain limits, can be perceived by the ear of the human being. If sounds can be produced by moving or striking parts in the case of some mechanical equipment, there are also structural transformations of certain metallic materials associated with generating sounds.

Objectives deriving from communication or entertainment equipment led to the appearance of audio speakers. Such loudspeakers convert electrical energy into mechanical energy that manifests in vibrations with characteristics corresponding to the sound field. Such loudspeakers have a fixed magnet near a coil through which an electric current flows, characterized by a variation in the intensity of the electric current in the audio frequency range. The electromagnet is solidarized with a flexible cone that is intended to amplify and direct the sounds. The interaction between the fields generated by the fixed magnet and the movable electromagnet drives the flexible material cone into motion, thus producing sounds.

As a rule, the passage of sounds through test samples made of different materials is studied using the so-called sound impedance tubes [18,24]. Indications regarding the measurement of sound absorption level when passing through different media have been included in two main standards regarding this field of research [25,26].

The equipment used to determine the sound absorption coefficient requires small samples in the form of discs inserted into the tube through which the sounds reflected by the walls of the cylindrical glass enclosure will also pass. A variant of the tube impedance is used in the two-microphone impedance method [12]. Maroo and Wright appreciated that the common impedance tube could not be used to determine sound transmission loss in the case of samples manufactured by 3D printing, especially in the case of high frequencies. For this reason, a small reverberation room was used [19]. Shtrepi and Prato showed that the currently used methods for studying the absorption of sounds by different materials have some disadvantages. For example, the impedance tube is less suitable for 3D systems, and a real reverberation chamber requires test pieces of large dimensions [27]. The research results completed through a doctoral thesis on designing a new reverberation chamber usable for characterizing sound transmission loss in the case of multi-material samples manufactured by 3D printing were published in 2021 by Maroo [28]. A reverberation chamber has been used by Peng et al. for the research of sound transmission loss in the case of some aircraft composite panels of large dimensions, which were not manufactured by 3D printing [29].

The content of this article is intended to highlight the behavior of small panels made of two polymeric materials by 3D printing when such panels are used as sound insulating materials. A finite element modeling was used to theoretically investigate the process of sound propagation through polymer panels. Later, using the results of some experimental research, empirical mathematical models were determined, and such models highlight the influence exerted by different factors on the acoustic pressure level of the sounds that passed through small panels made of 3D-printed polymer materials.

## 2. Materials and Methods

### 2.1. Propagation of Sounds through Panels Made of Polymeric Materials

**Figure 2:**
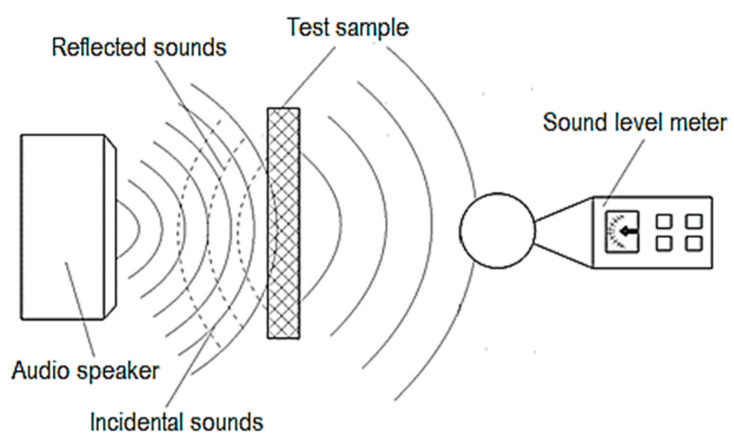
Evaluation of the characteristics of sounds after passing through a panel of polymeric material manufactured by 3D printing.

In the research framework, the results of which are presented in this paper, an audio mini speaker was used to generate sounds with variable frequencies and amplitudes (Figure 2). This audio mini speaker is model Bass, from Andowl, China, being a mini portable wireless speaker. The sounds generated by the audio mini speaker pass through a small panel made of polymer material. The polymer material panel was manufactured by 3D printing. Later, the sounds reach the sensor of a sound level meter, which will provide information about the characteristics of the sounds that have passed through the polymer material panel. The sound level meter is a digital instrument from Ckinnfon, model HY1361, Mainland, China. The three main components (the audio mini speaker, the polymer material panel, and the sound level meter) were placed in enclosures lined on the inside with a material capable of absorbing the energy of sound vibrations.

In principle, the velocity *v_g_* of sound in gases is considered to correspond to a shape relation:

(1) $$v_{g}=\sqrt{\frac{K}{\rho }},$$

where *K* is the bulk modulus and *ρ* is the gas density.

In the case of solid materials, the sound propagation speed can be evaluated using a shape relationship:

(2) $$v_{s}=\sqrt{\frac{E}{\rho }},$$

where *E* is the Young’s modulus and *ρ* is the density of the solid material.

In the case of macroporous polymers, the presence of the compressible air-filled pores contributes to a reduction of the longitudinal sound speed *v_l_*, reaching [30] values consistent with the equation:

(3) $$v_{l}=\sqrt{\frac{M}{\rho }},$$

where *M* is the longitudinal modulus, and *ρ* is the mass density of the porous material.

The speed of sounds in a body made of polymeric material can have different values, for example, from 1950 m/s in the case of polyethylene of low density to 2270 m/s in the case of polycarbonate and 2430 m/s in the case of polyethylene of high density.

Equations (1)–(3) highlight the fact that elements characterizing the nature and properties of the medium through which the sound waves propagate are capable of exerting an important influence on the energy carried by these waves and that measuring the sound energy can provide information on the capacity of different media to absorb, to a greater or lesser extent, the energy of the sound waves.

### 2.2. Experimental Conditions

The experimental research aimed to highlight the influence exerted by some factors on the reduction of the sound intensity level after passing the sounds through some small panels made of polymer materials by 3D printing.

In the case of the present research, we resorted to using equipment made up of several distinct compartments (Figure 2). It can be seen that there is a compartment in which the audio mini speaker was fixed. A second compartment allowed for the locating and clamping of the polymer sample through which the sound waves pass. Another compartment was where the sensor of a sound level meter was located. An intermediate compartment was provided to include the development of some experimental research on the influence of the distance between the audio mini speaker and the sensor of the sound level meter. The four compartments are made of wooden boards and assembled using wooden pins. To reduce the risk of the wooden walls of the premises influencing the intensity of sounds received by the sound level meter sensor, the interior surfaces of the compartments with wooden walls were covered with a porous polymeric material characterized by a high capacity of sound absorption.

**Figure 3:**
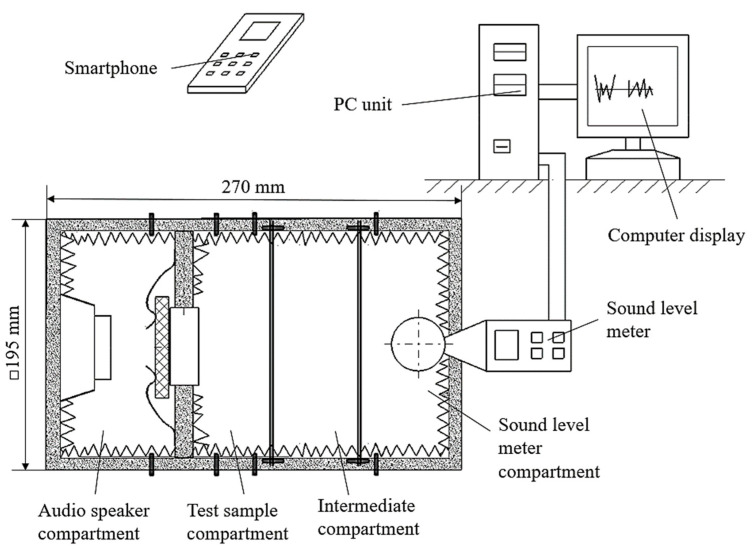
Schematic representation of the equipment used to highlight the intensity of the influence exerted by different factors on the sound pressure level after passing sounds through a panel of polymeric material manufactured by 3D printing.

**Figure 4:**
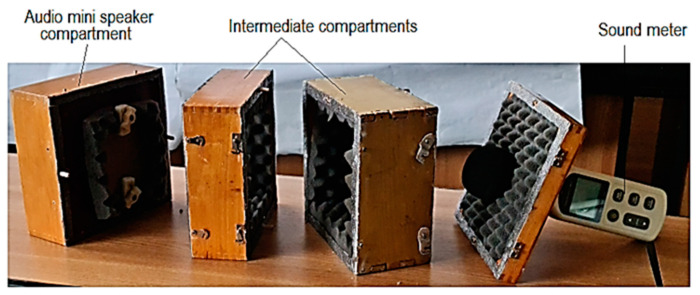
The appearance of some equipment components used to highlight the intensity of the influence exerted by different factors on the acoustic pressure level after the passage of sound waves through a panel of polymeric material manufactured by 3D printing.

An audio mini speaker type Andowl M10 was used (Figure 3 and Figure 4). The application use for phone control (using Bluetooth) of the mini audio speaker was the Frequency Generator built by LuxdeLux, being an online resource, specifically, an Android app, available on Play Store from Google.

The intensity of the influence exerted by different factors on an output parameter corresponding to the process of passing sounds through a small panel manufactured by 3D printing is dependent on the nature of the panel material, the thickness of its wall, some characteristics of the materials embedded in the panel that depends on the 3D printing conditions, the level of acoustic pressure and the frequency of the sound generated by a certain voltage source, etc.

Polylactic acid (PLA, produced by Filamentum, Fillamentum Manufacturing Czech s.r.o., Nám. Míru 1217, 768 24 Hulín, Czech Republic), high-impact polystyrene (HIPS, produced by Formfutura as EasyFil HIPS, Tarweweg 3, 6534 AM Nijmegen. The Netherlands), and polyethylene terephthalate glycol (PETG, produced by PrimaSelect as PrimaSelect™ PETG, PrimaCreator, Kantyxegatan 25 F, 213 76 Malmö, Sweden) were used as materials for the small panels. Some properties of the three materials are presented in Table 1. The three polymeric materials were chosen to be easily accessible and present different physical–mechanical properties. It was assumed that such materials would also have different sound insulation properties.

During the experimental tests, we noted some characteristics of the small panels (thickness and type of material), some parameters specific to the 3D printing process (being selected, in the present case, printing speed *v_p_*, infill percent *i*, infill pattern *i_p_*, and the thickness of the deposited layer *t*), and also some characteristics of the sounds generated by the audio mini speaker (frequency *f* and sound volume *s*).

The design of the experimental tests was carried out by considering an L18-type Taguchi factorial experiment, with seven input factors having three-level variance values and one input factor with two-level variation values. For the selection of the input factor with a two-level variation, it was assumed that an increase in the thickness *t* of the small panel would lead to a monotonous decrease (hence, one without maxima or minima) of the sound intensity, which would make it more appropriate to use only two levels of variation for the input factor (independent variable) mentioned.

Thus, the thickness *t* of the small panels (*t*_1_ = 1 mm and *t*_2_ = 5 mm) was selected as an input factor with values on two levels. The small panels had a surface with an area of 100 × 100 mm^2^. An Ultimaker 3 printer (Eindhoven, The Netherlands) was used for the 3D printing of panels from different polymer materials.

In the case of factors with values on three levels of variation, the nature of the material from the small panel (three distinct materials, these being the polylactic acid, which was assigned the value of *m_PLA_* = 1; high impact polystyrene HIPS, which was taken into account considering *m_HIPS_* = 2; and, respectively, polyethylene terephthalate glycol PETG, for which a symbol with the value *m_PETG_* = 3 was used), along with 3D printing speed (*v_p_*_1_ = 20 mm/s, *v_p_*_2_ = 40 mm/s, *v_p_*_3_ = 60 m/s), infill percentage *i* (*i*_1_ = 18%, *i*_2_ = 48%, *i*_3_ = 78%), infill pattern (*i_p_*_1_ for grid, *i_p_*_2_ for cubic, *i_p_*_3_ for gyroid), the thickness of the deposited layer (*t*_1_ = 0.05 mm, *t*_2_ = 0.1 mm, *t*_3_ = 0.15 mm), sound frequency (*f*_1_ = 5000 Hz, *f*_2_ = 10,000 Hz, *f*_3_ = 15,000 Hz), and sound volume *s*, expressed as a percentage of the maximum sound volume generated by the micro audio speaker used (*s*_1_ = 30%, *s*_2_ = 50%, *s*_3_ = 100%), were accounted for.

As output parameters from the analyzed process, the sound pressure level or sound pressure (this being a measure of the sound energy emitted by a sound source) was used. The unit of measure for evaluating the sound pressure level is the decibel, and it is the decimal logarithm of the ratio between the measured sound pressure and the reference sound pressure *p*_o_ = 2 × 10^−5^ Pa (the pressure that can be sensed by the human being, corresponding to a frequency of 1 kHz).

It is relatively common to measure acoustic pressure with the help of sound level meters.

The present research measured the acoustic pressure level using a sound level meter that can be connected to a computer. The use of specialized software allows us to highlight on the computer screen the evolution over time of the acoustic pressure level of the sound received under different conditions.

The values of the input factors (of the independent variables) were included in Table 2. For each input factor in the investigated process, the coded value according to the Taguchi methodology for an L18-type factorial experiment and the actual value of that factor were included. Also, control samples have been 3D printed considering the infill percentage parameter; it has been 6% and 96%, with the default type of infill assigned per percentage value. Each group of materials received two control samples. The results were consistent with the ones presented for the 18 samples.

## 3. Results

### 3.1. Modeling Sound Propagation through Polymer Panels Using the Finite Element Method

**Figure 5:**
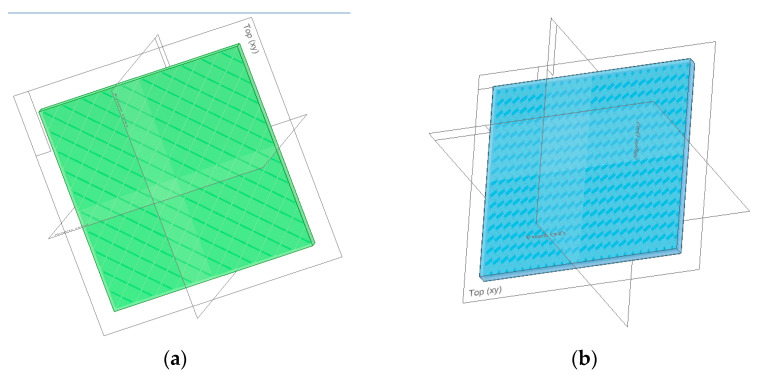
Graphical representations of designed polymeric panels: (**a**) panel with grid infill pattern; (**b**) panel with gyroid infill pattern.

The finite element method (FEM) offers a perspective on the way the polymeric panel deflects the sound and how it is propagated inside a closed enclosure. It needed the same infill pattern and thickness to provide accurate results as the analyzed 3D-printed panels. For that matter, the SolidEdge teacher edition was used to model two panels with different patterns, grids, and gyroid of 5 mm thick. They correspond to the R7 and R17 samples of 3D-printed parts for the experiments, having received the same infill percentage and shape (Figure 5). Each panel was later added to an assembly, including a speaker box and the panel itself at the same distance as the one from the experimental setup.

**Figure 6:**
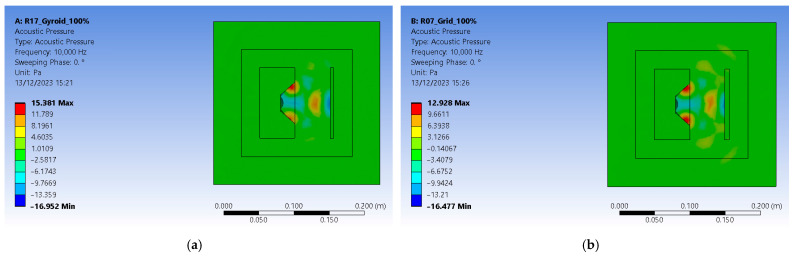
Graphical representations of acoustic pressure distribution: (**a**) setup with gyroid infill pattern; (**b**) setup with grid infill pattern.

Ansys 2023 R2 (researcher license), as the preferred software was chosen for FEM, with its module named Harmonic Acoustics, because of its ability to highlight a linear structure’s response to sinusoidal loads, which vary in time. That means that each boundary condition determines the frequency applied using its amplitude and phase. Different loads can be applied, from which the mass source was selected as the primary excitation method. It was necessary to model the acoustic medium surrounding each speaker with a panel type of assembly because this is the one computational domain (CD) in which acoustic waves propagate (Figure 6).

The authors acknowledge that further refinement may be needed and recommend using the information carefully.

### 3.2. Experimental Results

The experimental results were entered in the last columns of Table 2. Thus, in column no. 18, the values of the sound intensity level were mentioned when between the sound source (audio mini speaker) and the sensor of the sound meter level, which was not placed on a panel of polymeric material; and in column no. 19, the values were received by the sensor of the sound level meter when a panel of polymeric material with certain characteristics, obtained through the 3D printing process, was placed between the sound source and the sensor. In column no. 20, the differences between the values entered in the two previous columns were included. These values (from column no. 20) provide information on the extent to which the sound pressure level was affected by the presence of a polymeric material panel between the sound source and the sound-receiving sensor.

The experimental results were mathematically processed using specialized software based on the least squares method [31]. The software allows for the selection of a certain empirical mathematical model from among five such models (first-degree polynomial, second-degree polynomial, power-type function, exponential function, and hyperbolic function, respectively). The adequacy of a certain empirical mathematical model to the experimental results can be assessed using the value of the so-called Gauss criterion [32,33]. In principle, the value of Gauss’s criterion is determined as a ratio in which the numerator is a sum of the squares of the differences between the values determined by using the proposed empirical mathematical model and, respectively, the values of the ordinates corresponding to the experimental results, for the same values of the abscissas. The ratio’s denominator represents the difference between the number of experimental trials and the number of constants in the proposed empirical mathematical model. It is considered that the lower the value of Gauss’s criterion, the more appropriate the considered mathematical model is for the set of experimental results obtained. According to this observation, it can be appreciated that when several empirical mathematical models are proposed, the most appropriate model will be the one for which the value of Gauss’s criterion is the lowest.

Through the mathematical processing of the experimental results, it was found that the most appropriate empirical mathematical models for the sets of experimental results are the following:

- For the absolute values determined in the case of the sound pressure level, it is a polynomial function of the second degree:
  $$\begin{array}{ll} p_{ac} & =61.589+12.355t-1.944t^{2}-2.859m+0.941m^{2}-0.117v\\ & +0.00153v^{2}-0.321i+0.00315i^{2}+0.815i_{p}+0.216i_{p}^{2}\\ & +89.372l-444.032l^{2}+0.00853f-4.351\cdot 10^{-9}f^{2}\\ & +0.825s-0.00241s^{2},\; \end{array}$$
  for which the value of Gauss’s criterion is *S_G_* = 0.3702504;
- For the sound pressure level corresponding to the energy absorbed primarily by the panel material manufactured by 3D printing, calculated as a difference between the values entered in columns 18 and 19 of Table 2, the most appropriate empirical mathematical model is like a polynomial function of the second degree:
  $$\begin{array}{ll} \Delta p_{ac}= & 169.745-263.917t+43.719t^{2}+\\ & 5.097m-1.639m^{2}-0.141v+0.00153v^{2}+\\ & 0.180i-0.00177i^{2}+1.311i_{p}-0.571i_{p}^{2}-\\ & 27.377l+222.774l^{2}+0.00853f-3.253\cdot 10^{-7}\cdot f^{2}+\\ & 0.685s-0.00480s^{2}, \end{array}$$
  the value of Gauss’s criterion being, in this case, *S_G_* = 0.4655038.

On the other hand, in manufacturing engineering, empirical mathematical models of the power function type have been used for a relatively long time. Such models are used, for example, to highlight the influence of the cutting parameters on the size of the cutting tool life, the sizes of the cutting forces, the value of the roughness parameters, etc. It is considered that by simply examining the values of the exponents attached to the independent variables in a power-type function mathematical model, some first information is obtained relatively quickly regarding the intensity and sense of the influence exerted by the action of the input factors (consider as independent variables) in the process investigated on a magnitude of interest. By resorting also in the present case to such empirical mathematical models of the power function type, the following relations were determined:

- For the absolute values of the acoustic pressure level (values entered in column no. 19 of Table 2):
  $$p_{ac}=1198.755t^{-0.000324}m^{-0.0175}v^{-0.0314}i^{-0.0348}i_{p}^{0.0654}l^{0.0406}f^{-0.460}s^{0.419},$$
  the value of Gauss’s criterion being higher than that of the most appropriate model, namely, *S_G_* = 28.49857.
- For the acoustic pressure level corresponding to the energy absorbed by the panel material:
  $$\Delta p_{ac}=6.726\cdot 10^{-6}t^{-0.121}m^{-0.0904}i^{0.0322}i_{p}^{-0.0939}l^{0.113}f^{1.618}s^{0.102},$$
  for which the value of Gauss’s criterion has value *S_G_* = 56.99927.

**Figure 7:**
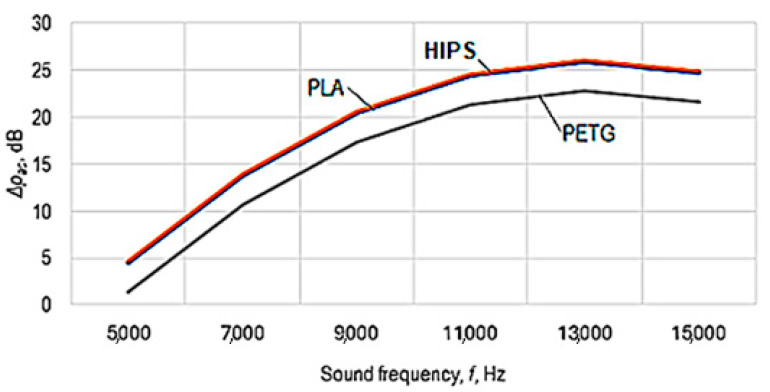
The influence exerted by the frequency *f* of the sounds and by the type *m* of the panel material of polymer material manufactured by 3D printing on the difference in acoustic pressure Δ*p_ac_* (panel thickness *t* = 5 mm; printing speed *v* = 60 mm/s; infill percent *i* = 48%; infill pattern: cubic (*i_p_* = 2); layer thickness *l* = 0.06 mm; sound volume *s* = 100%).

**Figure 8:**
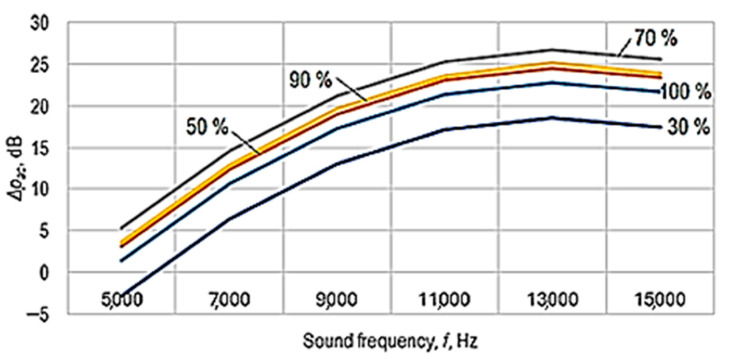
The influence of sound frequency *f* on the difference in acoustic pressure for different sound volume values (panel thickness *t* = 5 mm; test piece material: PETG (*m* = 3); printing speed *v* = 60 mm/s; infill percent *i* = 48%; infill pattern: cubic (*i_p_* = 2); layer thickness *l* = 0.06 mm; empirical mathematical model of the second degree polynomial type).

**Figure 9:**
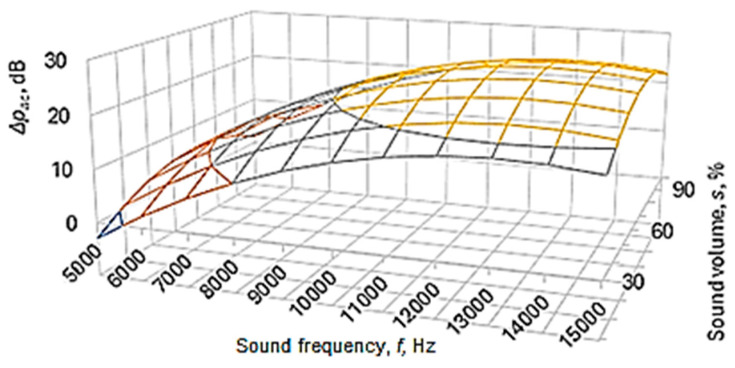
The influence of sound frequency *f* and sound volume on the difference in acoustic pressure Δ*p_ac_* (test piece material PETG (*m* = 3); printing speed *v* = 60 mm/s; infill percent *i* = 48%; infill pattern: cubic (*i_p_* = 2); layer thickness *l* = 0.06 mm; empirical mathematical model of the second-degree polynomial type).

The graphical representations in Figure 7, Figure 8 and Figure 9 were drawn using Equation (5).

## 4. Discussion

The validation of the empirical mathematical model proposed by Equation (5) can be performed by making an additional experimental test using for one independent variable or for several independent variables, other values different from those used to generate the empirical mathematical models, by processing the experimental results. In the case of the mathematical model represented by Equation (5), an additional experimental test was performed. Experiment no. 18 from Table 2 was taken into account. The following values of the independent variables were used: *t* = 5 mm; *m* = 3 (corresponding to PETG material); *v* = 60 mm/s; *i* = 48%; *i_p_* = grid; *l* = 0.1 mm; *f* = 8000 Hz; and *s* = 7500 Hz. It can be seen that the last two independent variables (*f* și *s*) had values other than those used to determine the empirical mathematical model corresponding to Equation (5) in the case of experiment no. 18.

For these independent variables’ values, the Δ*p_ac_* parameter value, determined using the empirical mathematical model (5), was 17.31 dB. Through the experimental test, the values *I*_0_ = 117.5 dB were obtained without the panel and, respectively, *I_p_* = 101.2 dB when the PETG panel was placed between the mini audio speaker and the sensor of the sound level meter. The difference between the two measured values leads to a value of Δ*p_ac_* = 16.3 dB. It is thus possible to determine a percentage difference between the value determined using the identified empirical mathematical model and the value obtained using the experimental result of the additional test Δ = (17.31 − 16.3) × 100/17.31 = 5.83%. It can thus be appreciated that the proposed empirical mathematical model can be considered validated.

The analysis of Equations (6) and (7)—and, respectively, of the graphic representations in Figure 7, Figure 8 and Figure 9—facilitated the formulation of the following findings.

From the analysis of the power-function-type mathematical models (Equations (6) and (7)), it is found that the greatest influences on the values of the acoustic pressure level *p_ac_*—and, respectively, on the sound energy absorption highlighted by the acoustic pressure difference Δ*p_ac_*—correspond to the factors frequency *f* and sound volume *s*, as these factors are associated, in Equations (6) and (7), with relatively high absolute values of exponents (in Equation (7), the two magnitudes are associated with exponent values of 1.618 in the case of frequency *f* and, respectively, 0.102 in the case of sound volume *s*).

The empirical mathematical model of power function type defined by Equation (7) highlights the strong influence of frequency *f* and sound volume *s* on the sound energy absorbed by the sample material.

Using mathematical models of the second-degree polynomial type, the graphic representations in Figure 7, Figure 8 and Figure 9 highlight the existence of maxima of the value of the acoustic pressure difference Δ*p_ac_*. The more pronounced influence of the frequency *f* of the sounds exerted on the difference in acoustic pressure level can be more clearly observed in the graphic representation in Figure 9.

For the conditions in which the experimental research was carried out, from Figure 7, Figure 8 and Figure 9, it can be seen that there is a maximum of the acoustic pressure difference for a frequency of about 13,000 Hz. It is worth noting that different researchers have observed maximum sound absorption values by panels made of different materials for certain frequency values. These maximum values differ depending on the nature of the panel material and other conditions in which the measurements were made. Thus, it was found the existence of a maximum absorption coefficient for a frequency of approximately 2000 Hz in the case of a panel made of 20% polyester resin and 80% PET pellets [6]. Others highlighted the existence of two maxima of the sound absorption coefficient for frequencies with approximate values of 2500–4000 Hz and, respectively, of approximately 7500–9500 Hz in the case of panels made of porous solid fiber structures [9].

As mentioned in the introduction, there is an influence exerted on the sound absorption capacity in the case of some panels of polymeric materials by some input factors in the experimental investigation process, such as the sound frequency [6,8,9,12,14,15,20,22], the nature of the materials from which the samples were made [3,6,7,8,9], the acoustic pressure level of the incident sound radiation [5], the thickness of the panels [3,5,13,15,23], the structure corresponding to the arrangement of the material in samples [2,3,9,10,11,12,13,14,15,16,17,18,19,20] etc. However, the values that characterize the sound energy absorption by the samples have values between wide limits due to the differences corresponding to the conditions involved in making the measurements. Through the experimental results presented in this paper, as expected, it is confirmed that an increase in the thickness *t* of the panel will lead to the absorption of a greater amount of the energy of the sounds emitted by the source.

**Figure 10:**
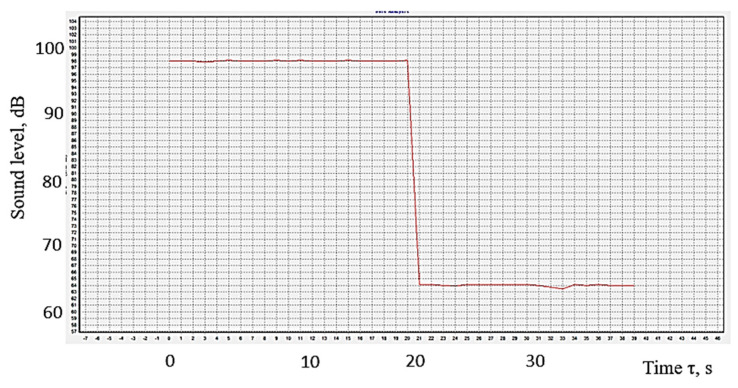
The difference in sound level (acoustic pressure) in the case of the absence of the panel and the presence of the polymer panel manufactured by 3D printing for experiment no. 2.

Figure 10 shows the reduction of the sound level when a panel made of polymeric material manufactured by 3D printing was placed between the sound source and the sensor of the equipment under the conditions valid for experiment no. 2. As expected, the presence of the polymer panel contributes to a decrease in the sound level received by the device’s sensor. It can be noted that the panel made of polymer material absorbs energy corresponding to a difference in acoustic pressure of about 35 dB. This means that the presence of the polylactic acid panel leads to a reduction of about 45% in the sound pressure level.

**Figure 11:**
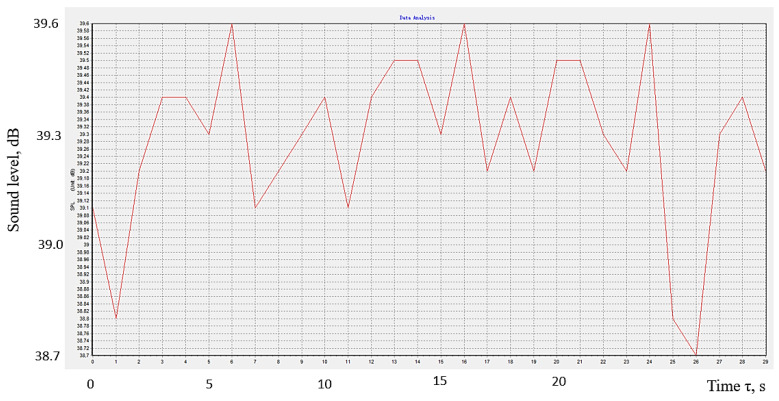
The time variation of the sound level when using the panel made of polymeric material manufactured by 3D printing in experiment no. 18 (the points correspond to the mean values of the sound level recorded for a duration of one second).

The graphic representation in Figure 11 sought to highlight the time variation of the sound level reaching the sound level meter sensor. The graph corresponds to the use of the conditions corresponding to experiment no. 18. The sound level meter transmits the values to the computer software, which determines a mean sound level value after one-second intervals. Such mean values were considered when the computer software developed the diagram in Figure 11. It can be seen that the sound level reaching the sound level meter sensor after passing through the polymer material panel is not constant. For the conditions of the experimental tests, the sound level registers a variation between relatively close limits, namely, between 38.7 dB and 39.6 dB.

**Figure 12:**
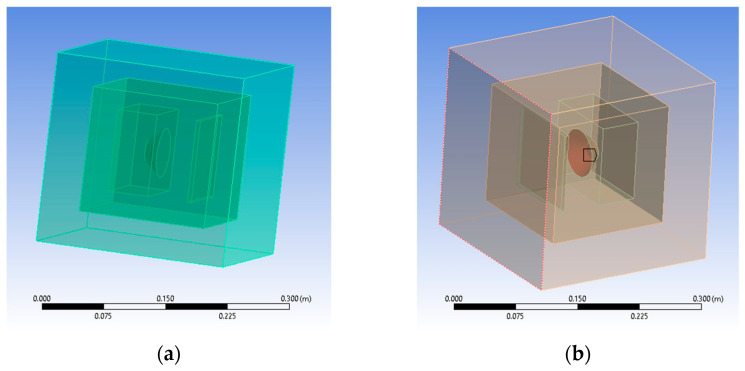
Graphical representations setup with two enclosures: (**a**) general view of resulted geometry; (**b**) mass source excitation applied to the speaker’s membrane.

To simulate a perfect insulation environment, another medium had to be modeled as a perfectly matched layer (PML) which absorbs bouncing waves without reflecting them. In the Ansys Space Claim module, the boxed type of enclosure was chosen was for both media—computational domain (CD) and the perfectly matched layer (PML)—which were later imported in the analysis’s module (Figure 12a). The entire geometry shares its topology among its components. Because the aim is to obtain acoustic pressure distribution results, air was added as the propagation medium and assigned as the default material for both enclosures. Mesh-wise, a face-sizing method was added to capture all facets of the newly designed assembly by suppressing the initial setup containing the speaker and the polymeric panel. That made the assignation of materials for the two-suppressed components irrelevant. Mesh resulted in 60,553 nodes and 38,745 elements. The mass excitation source was applied to the speaker’s membrane (Figure 12b). When applied to a surface, one can divide the mass flow rate by the area on which it is applied. After this step, the speaker and polymeric panel are suppressed to mesh the entire geometry.

The analysis settings received a linear frequency spacing varying from zero to 10,000 Hz. The acoustic region is set to the DM enclosure, and a newly added physics region is added and assigned to the PML enclosure. This ensures that pressure builds up inside as acoustic waves do not escape the setup and are not reflected by its outer boundaries. The solution requires a sound pressure level and an acoustic pressure distribution. For the R17 experiment, the gyroid 18% infilled sample at 100% sound intensity level resulted in 119.66 dB (Figure 12b). For the R7 experiment, the grid 48% infilled sample at 100% sound intensity level resulted in 119.01 dB (Figure 12b). The values are greater than the ones recorded in experimental tests since Ansys uses a perfectly isolated medium.

**Figure 13:**
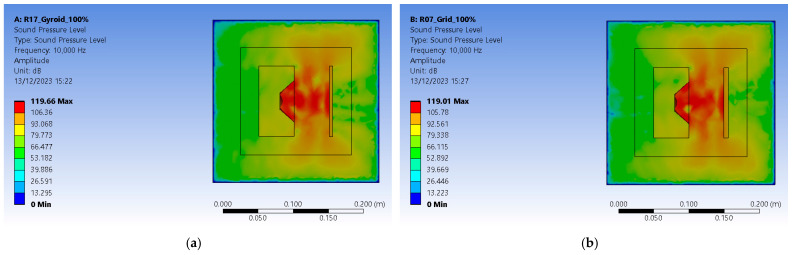
Graphical representations of sound pressure levels: (**a**) setup with gyroid infill pattern; (**b**) setup with grid infill pattern.

Acoustic pressure builds up inside the computational domain (CD) and extends to the limits of PML enclosure. The R17 experiment setup peaks at 15.381 Pa, and the R7 experiment reaches 12.928 Pa (Figure 13). Results show that gyroid infill better deflects the sound waves. They are consistent with experimental results that revealed a 26.6 dB loss in sound pressure level for the R7 experiment gyroid-infilled sample instead of just 16.8 dB for the R17 experiment grid-infilled one.

## 5. Conclusions

In some situations, reducing the intensity of sounds in certain spaces is necessary. In such situations, panels made of polymeric materials can be used to allow for the sound insulation of the respective spaces. For this reason, it is useful to know some information about the ability of some polymeric materials to absorb sound energy. On the other hand, the expansion of the use of parts made of polymeric materials through 3D printing has opened up new possibilities for modification, including the ability of some polymeric materials to absorb the energy of sound vibrations. The finite element method was employed to obtain initial information on the behavior of small polymeric-med panels. Later, experimental research was carried out, starting with a Taguchi L18-type factorial experiment. The experiment considered eight independent variables, of which one variable (the thickness of the polymer material panel) received values on two levels of variation. The other seven independent variables had values on three levels of variation. The input factors were the thickness of the panel, the nature of the polymer material of the panel, the 3D printing speed, the infill percentage, the infill pattern, the layer thickness, the frequency, and the sound volume. The output parameters taken into account as dependent variables were the acoustic pressure level of the sound wave after passing through the polymer material panel and, respectively, the acoustic pressure level corresponding to the energy absorbed by the panel. The experimental research was carried out using a sound-insulated enclosure. In this enclosure, in separate compartments, a mini audio speaker, a supporting wall of the sample in the form of a small panel, and, respectively, the sensor of a sound level meter were placed. Panels with an area of 100 × 100 mm^2^ and 5 mm thicknesses were manufactured by 3D printing from polylactic acid and polyethylene terephthalate glycol, respectively. The experimental results were mathematically processed using specialized software. Empirical mathematical models of the second-degree polynomial and power function were obtained in this way. The last type of function directly highlights the means and intensity of the influences exerted by the input factors on the values of the output parameters. As expected, it was found that an increase in the thickness of the polymer material panel leads to a decrease in the sound pressure level after sound passes through the polymer material panel. The strongest influence on the difference in acoustic pressure is due to the energy the panel absorbs, which is exerted by the frequency of the sounds. For the polymer materials used to make the small panels and for the conditions of the experimental tests, the variation of the acoustic pressure difference due to the change in the frequency of the sounds led to a maximum corresponding to a frequency of about 13,000 Hz. In the future, research is intended to continue considering the possible influence exerted by other factors that characterize the 3D printing process. Another direction for experimental research could consider panels manufactured by 3D printing from other materials.

## Figures and Tables

**Table 1:** Initial properties of materials used for the 3D printing of small panels.

Material	Properties		
	Density, *ρ*, kg/m^3^	Tensile Strength, *Rm*, MPa	Printing Temperature, °C
Polylactic acid (PLA)	1250	32.9	180–220
High-impact polystyrene (HIPS)	1040	42	220–240
Polyethylene terephthalate glycol (PETG)	1230	31.9	220–260

**Table 2:** Values of input factors considered and experimental results.

Exp. No.	Input Factors																A-Coustic Pressure Level without Panel, *I*_0_, dB	A-Coustic Pressure Level When Using Polymer Panel, *I_p_*, dB	Difference in A-Coustic Pressure Level, Δ*p_ac_*, dB
	Panel Thickness, *t*		Material Type, *m*		Printing Speed, *v*		Infill Percent, *i*		Infill Pattern, *i_p_*		Layer Thickness, *l*		Sound Frequency, *f*		Sound Volume, *s*				
	Coded Value	Real Value, mm	Coded Value	Real Value	Coded Value	Real Value, mm/s	Coded Value	Real Value, %	Coded Value	Real Value	Coded Value	Real Value, mm	Coded Value	Real Value, Hz	Coded Value	Real Value, %			
Column No. 1	2	3	4	5	6	7	8	9	10	11	12	13	14	15	16	17	18	19	20
R1	1	1	1	PLA	1	20	1	18	1	Grid	1	0.06	1	5000	1	30	77.7	72.8	4.9
R2	1	1	1	PLA	2	40	2	48	2	Cubic	2	0.1	2	10,000	2	50	95.8	65.8	30
R3	1	1	1	PLA	3	60	3	78	3	Gyroid	3	0.15	3	15,000	3	100	103.6	74.6	29
R4	1	1	2	HIPS	1	20	1	18	2	Cubic	2	0.1	3	15,000	3	100	103.6	75.7	28.4
R5	1	1	2	HIPS	2	40	2	48	3	Gyroid	3	0.15	1	5000	1	30	77.7	73.0	4.7
R6	1	1	2	HIPS	3	60	3	78	1	Grid	1	0.06	2	10,000	2	50	95.8	67.7	28.1
R7	1	1	3	PETG	1	20	2	48	1	Grid	3	0.15	2	10,000	3	100	115.5	88.9	26.6
R8	1	1	3	PETG	2	40	3	78	2	Cubic	1	0.06	3	15,000	1	30	58.5	38.6	19.9
R9	1	1	3	PETG	3	60	1	18	3	Gyroid	2	0.1	1	5000	2	50	95.9	91.8	4.1
R10	2	5	1	PLA	1	20	3	78	3	Gyroid	2	0.1	2	10,000	1	30	77.7	61.5	16.2
R11	2	5	1	PLA	2	40	1	18	1	Grid	3	0.15	3	15,000	2	50	78.1	52.1	26
R12	2	5	1	PLA	3	60	2	48	2	Cubic	1	0.06	1	5000	3	100	114.5	110.5	4
R13	2	5	2	HIPS	1	20	2	48	3	Gyroid	1	0.06	3	15,000	2	50	78.1	51.8	26.3
R14	2	5	2	HIPS	2	40	3	78	1	Grid	2	0.1	1	5000	3	100	114.5	110.2	4.3
R15	2	5	2	HIPS	3	60	1	18	2	Cubic	3	0.15	2	10,000	1	30	77.7	58.7	19
R16	2	5	3	PETG	1	20	3	78	2	Cubic	3	0.15	1	5000	2	50	95.9	90.7	5.2
R17	2	5	3	PETG	2	40	1	18	3	Gyroid	1	0.06	2	10,000	3	100	115.5	98.7	16.8
R18	2	5	3	PETG	3	60	2	48	1	Grid	2	0.1	3	15,000	1	30	58.5	39.5	19

## Data Availability

All information necessary to understand the authors’ considerations has been included in the article. Other information will be provided by the authors of the article at the request of those interested in such other information.
